# Don't Wait- Ablate! Catheter Ablation in Patients with Advanced Heart Failure and Atrial Fibrillation

**DOI:** 10.1007/s11897-026-00787-4

**Published:** 2026-10-27

**Authors:** Maximilian Moersdorf, Philipp Sommer, Christian Sohns

**Affiliations:** 1 Department of Electrophysiology, Heart- and Diabetescenter NRW, Ruhr-University Bochum, Bad Oeynhausen, Germany; 2 Tulane Research Innovation for Arrhythmia Discovery (TRIAD), Tulane University, New Orleans, LA USA; 3 Department of Electrophysiology, Heart- and Diabetescenter NRW, University Bielefeld, Medical Faculty OWL, Bad Oeynhausen, Germany

**Keywords:** Advanced heart failure, Atrial fibrillation, Catheter ablation, Heart transplantation, Mortality

## Abstract

**Purpose of Review:**

Heart failure (HF) and atrial fibrillation (AF) frequently coexist and mutually accelerate disease progression through complex hemodynamic, neurohormonal, and structural interactions. Although earlier rhythm-control strategies failed to demonstrate mortality benefit, largely due to limitations of antiarrhythmic drug therapy, catheter ablation has fundamentally altered the therapeutic landscape.

**Recent Findings:**

The CASTLE-AF trial established catheter ablation as a disease-modifying therapy in patients with AF and HF with reduced ejection fraction (HFrEF), demonstrating reductions in mortality and HF hospitalization. More recently, the CASTLE-HTx trial extended these findings to patients with end-stage HF eligible for heart transplantation (HTx) or left ventricular assist device (LVAD) therapy. Catheter ablation has demonstrated a marked reduction in mortality and the need of an urgent HTx, or LVAD implantation compared with medical therapy alone. Subsequent subanalyses further refined risk stratification and identified patient subsets deriving the greatest benefit. Therefore, catheter ablation has received a class 1 recommendation for patients with AF and HFrEF.

**Summary:**

According to recent clinical evidence supporting early rhythm control through catheter ablation in advanced HF, it leads to a paradigm shift in the management of this high-risk population: in selected patients with AF and advanced HF, clinicians should not delay referral—“Don’t wait, ablate.”

## Introduction

Atrial fibrillation (AF) is the most common sustained arrhythmia worldwide, affecting more than 33 million individuals globally [1]. Heart failure (HF) is similarly prevalent and represents a major cause of morbidity and mortality [2]. AF and HF frequently coexist, with AF present in up to one-third of patients with chronic HF and in more than half of patients with newly diagnosed HF [3]. Importantly, AF is associated with increased hospitalization, stroke, and mortality rates across all HF phenotypes, including HF with reduced ejection fraction (HFrEF) and HF with preserved ejection fraction (HFpEF) [4].

Historically, rhythm control in AF, primarily achieved through antiarrhythmic drugs (AAD), failed to demonstrate survival benefit in large randomized trials such as AFFIRM and AF-CHF [5, 6]. Consequently, rate control became the dominant strategy. However, catheter ablation was shown as an effective rhythm control modality with the potential to modify disease progression. The CASTLE-AF trial demonstrated that catheter ablation in patients with AF and HFrEF significantly reduced mortality and HF hospitalization compared with medical therapy alone [7]. More recently, the CASTLE-HTx trial extended this paradigm into the most advanced HF population, patients who were eligible for heart transplantation (HTx), demonstrating a significant reduction in mortality, and the need for left ventricular assist device (LVAD) implantation, or urgent HTx after undergoing catheter ablation [8]. A subsequent subanalysis further elucidated risk profiles and treatment response [9]. This review integrates mechanistic rationale and clinical evidence to support early and proactive catheter ablation in patients with advanced HF and AF.

## Pathophysiological Interplay Between AF and HF

**Fig. 1:**
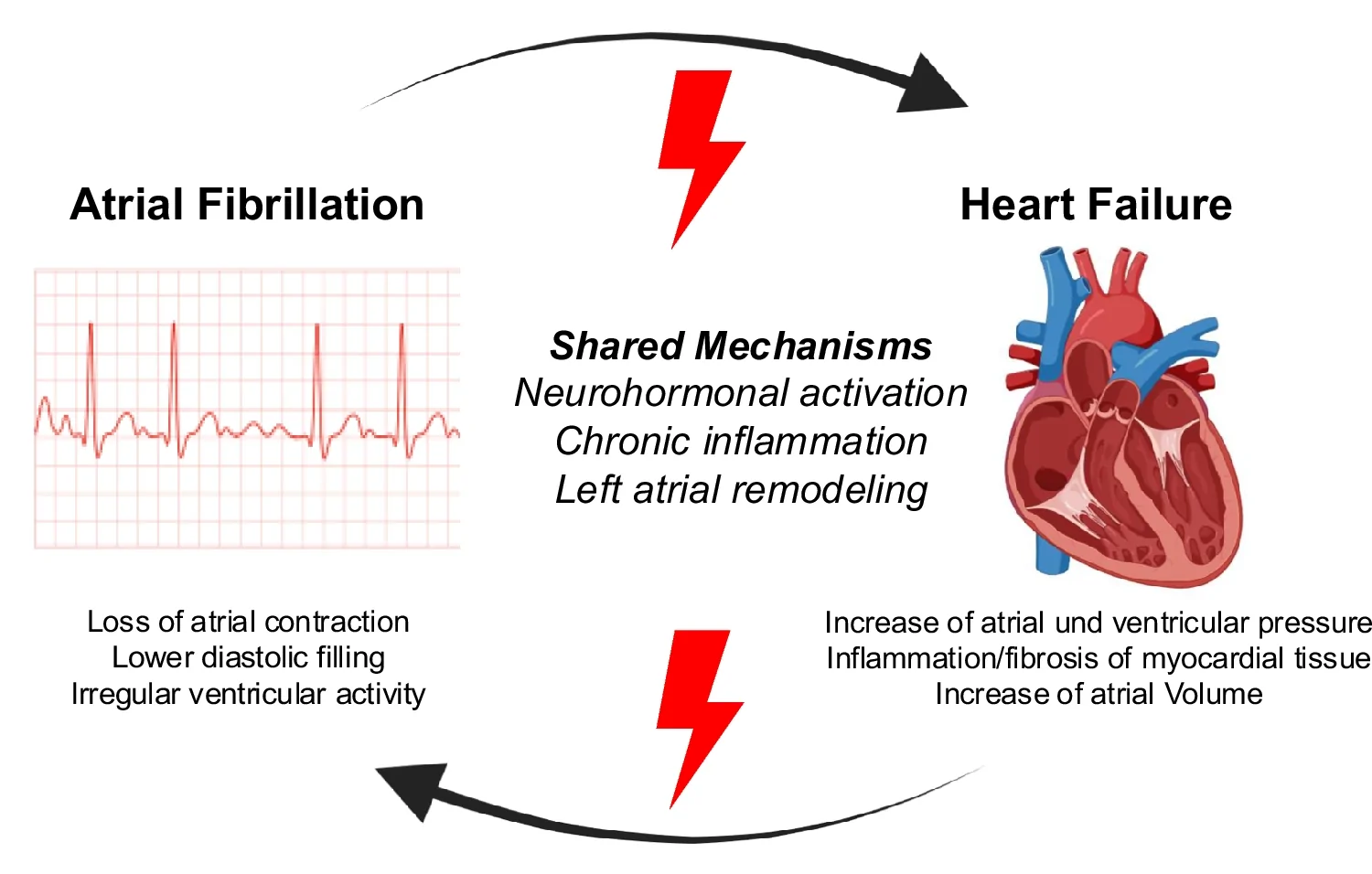
**Atrial fibrillation and heart failure – a bidirectional relationship.** Bidirectional pathophysiological interaction between atrial fibrillation (AF) and heart failure (HF). AF contributes to HF progression through loss of atrial contraction, impaired diastolic filling, and irregular ventricular activation, resulting in reduced cardiac efficiency and hemodynamic deterioration. Conversely, HF promotes the development and maintenance of AF through increased atrial and ventricular filling pressures, myocardial inflammation and fibrosis, and progressive atrial remodeling and enlargement. Both disease entities share common mechanistic pathways, including neurohormonal activation, chronic inflammation, and structural atrial remodeling, thereby creating a self-perpetuating vicious cycle that accelerates disease progression and worsens clinical outcomes

AF contributes to HF progression through several interrelated mechanisms (Fig. 1). The loss of atrial systole reduces ventricular filling and stroke volume, particularly in patients with impaired ventricular compliance [10, 11]. In HFrEF, where ventricular function is already compromised, this reduction in preload may significantly impair cardiac output. Additionally, the irregular ventricular response characteristic of AF leads to beat-to-beat variability in stroke volume, reducing overall hemodynamic efficiency independent of mean heart rate [12]. Furthermore, sustained tachycardia may result in tachycardia-induced cardiomyopathy, a reversible condition characterized by left ventricular systolic dysfunction [13–15]. Beyond hemodynamic effects, AF promotes neurohormonal activation, including the renin–angiotensin–aldosterone system (RAAS) and sympathetic nervous system, both of which contribute to myocardial fibrosis, adverse remodeling, and progressive ventricular dysfunction [16]. Structural remodeling extends beyond the ventricle. Atrial dilation and fibrosis contribute to functional mitral regurgitation, further increasing left atrial pressure and ventricular volume overload [17]. These processes establish a vicious cycle in which AF accelerates HF progression [18].

Conversely, HFrEF promotes AF development through elevated filling pressures, atrial stretch, and neurohormonal activation. RAAS-mediated pathways, including TGF-β signaling, promote atrial fibrosis and conduction heterogeneity, facilitating AF initiation and maintenance [16]. The bidirectional interaction between AF and HFrEF underscores the importance of early rhythm control strategies aimed at interrupting this cycle.

## Evolution of Rhythm Control in HFrEF

**Fig. 2:**
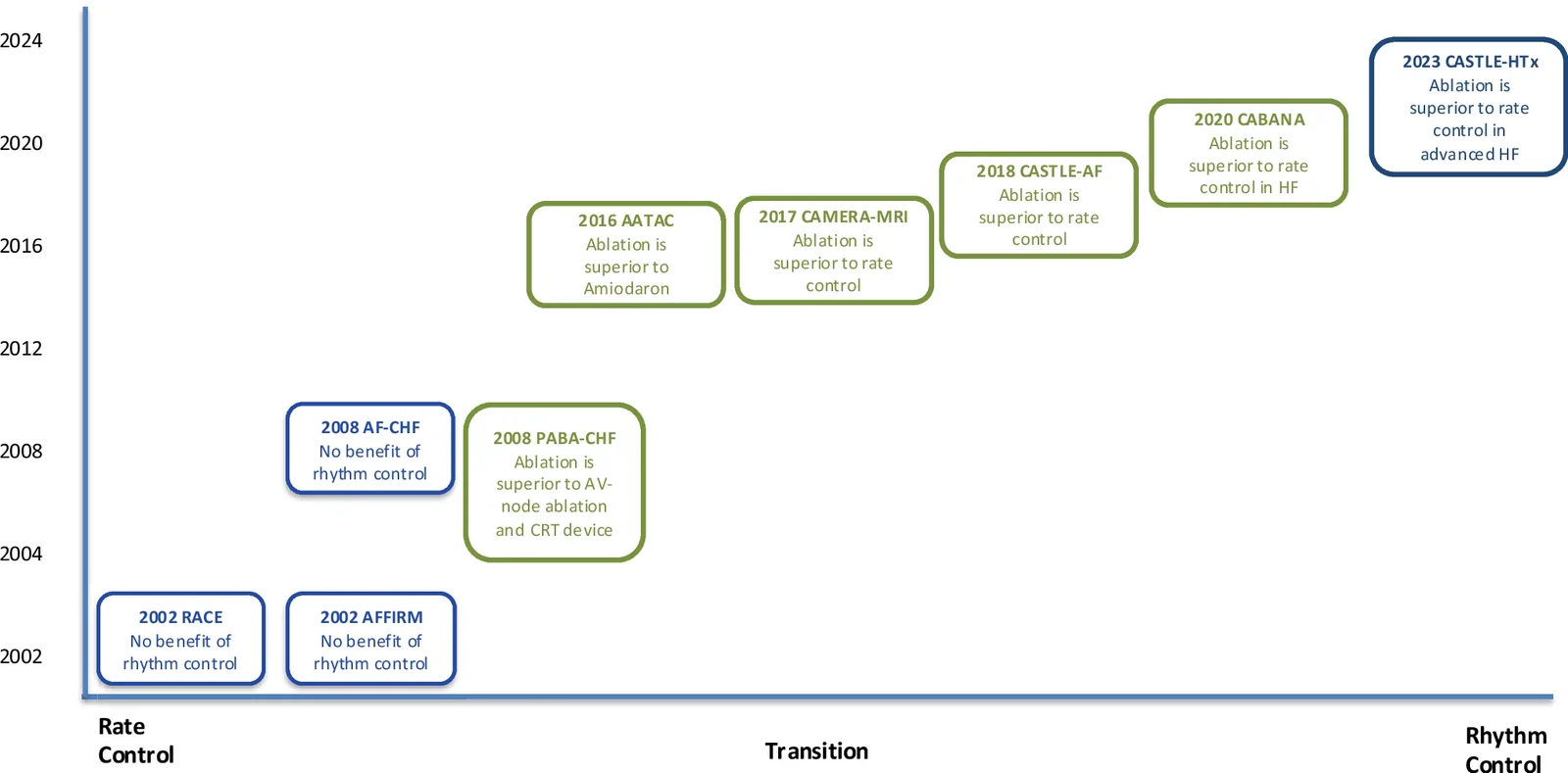
**Evolution in atrial fibrillation therapy: from rate to rhythm control.** Evolution of evidence supporting the transition from rate control to rhythm control strategies in atrial fibrillation (AF). The y-axis represents publication year, whereas the x-axis illustrates the conceptual shift from predominantly ratecontrol approaches toward rhythm control and catheter ablation-based strategies. Early landmark trials, including RACE Trial, AFFIRM Trial, and AF-CHF Trial, demonstrated no significant survival advantage of pharmacological rhythm control over rate control. Subsequent catheter ablation studies, including PABA-CHF Trial, AATAC Trial, CAMERA-MRI Trial, and CASTLE-AF Trial, established the prognostic and functional benefits of catheter ablation, particularly in patients with heart failure. More recent studies, including CABANA Trial, and CASTLE-HTx Trial, further supported earlier rhythm control strategies and extended the concept of AF ablation as a disease-modifying therapy across the spectrum of heart failure severity

Early rhythm control strategies were limited by the modest efficacy and adverse effects of AADs as described in the AFFIRM and RACE trials [5, 19]. In AF-CHF, AF and HF patients with an left ventricular ejection fraction (LVEF) of less than 35% were included [6]. It compared outcomes related to stroke, worsening HF events, and mortality between those receiving rhythm control and those on rate control. Rhythm control, primarily achieved due to amiodaron usage (82%), did not reduce the endpoints including mortality compared with rate control (HR 1.06; *P* = 0.59). These findings contributed to the longstanding preference for rate control. However, mechanistic studies suggested that maintenance of sinus rhythm could improve ventricular function if achieved effectively. The PABA-CHF trial showed that catheter ablation improved functional capacity (Six-minute walk distance test 340 m vs. 297 m, *p* < 0.001) and ventricular function (LVEF 35% vs. 28%, *p* < 0.001) compared with AV node ablation and cardiac resynchronization therapy in patients with a mean LVEF of less than 40% [20]. These observations provided the basis for randomized outcome trials evaluating catheter ablation in HFrEF (Fig. 2). More recently, emerging technologies such as pulsed field ablation (PFA) may represent the next step in rhythm control for HF patients by enabling faster procedures with improved tissue selectivity and potentially lower periprocedural risk in this vulnerable population [21].

## Catheter Ablation in HFrEF

### CASTLE-AF

The CASTLE-AF trial provided the first definitive evidence that catheter ablation confers a prognostic benefit in patients with AF and HFrEF [7]. In this randomized trial, patients with symptomatic AF, LVEF ≤ 35%, and implanted cardiac device were assigned to catheter ablation plus guideline-directed medical therapy (GDMT) or GDMT alone. The primary endpoint, a composite of all-cause mortality or HF related hospitalization, was significantly reduced in the ablation group (28.5% vs 44.6%; *P* = 0.007). Notably, catheter ablation resulted in a 47% relative reduction in all-cause mortality (Hazard ratio (HR) 0.53; 95%CI 0.32–0.86; *P* = 0.01), alongside a significant reduction in HF hospitalizations.

Beyond these primary findings, CASTLE-AF offered important mechanistic insights into the relationship between AF burden and clinical outcomes. Continuous rhythm monitoring via implanted devices enabled precise quantification of AF burden over time. A subsequent subanalysis demonstrated that reduction in AF burden to < 50% after 6 months was associated with improved outcomes [22]. However, a critical observation was that the survival benefit was driven almost exclusively by patients undergoing catheter ablation. In contrast, patients managed with medical therapy alone did not experience a comparable reduction in mortality, even when AF burden was reduced below this 50% threshold.

This finding suggests that the prognostic benefit of catheter ablation cannot be explained solely by AF burden reduction. Rather, ablation may exert additional effects beyond arrhythmia suppression, including more consistent rhythm control, improved ventricular rate regularity, and favorable modulation of neurohumoral and structural remodeling. Furthermore, catheter ablation may achieve more durable and stable reductions in AF burden compared with pharmacological strategies, which are often limited by incomplete efficacy and variability over time.

From a clinical standpoint, these data reinforce the role of catheter ablation as a disease-modifying intervention rather than a purely symptomatic therapy. The differential impact on mortality between ablation and medical therapy—despite similar AF burden thresholds in some patients—supports the notion that ablation provides unique mechanistic advantages.

In summary, CASTLE-AF established catheter ablation as a cornerstone therapy in patients with AF and HFrEF, demonstrating not only reductions in hospitalization but also a robust survival benefit. The subanalysis linking AF burden reduction to outcomes further highlights that catheter ablation delivers benefits beyond simple arrhythmia suppression, strengthening the rationale for early and proactive rhythm control strategies in this population.

### AATAC, CAMERA-MRI, and CABANA

Beyond CASTLE-AF, additional randomized data from the AATAC and CABANA trials provide important context supporting the role of catheter ablation in patients with AF and HFrEF, particularly with regard to rhythm control efficacy, clinical outcomes, and generalizability across different patient populations. The AATAC trial directly compared catheter ablation with amiodarone in patients with persistent AF and HFrEF (LVEF ≤ 40%), all of whom had implanted devices allowing continuous rhythm monitoring [23]. Over a follow-up period of 24 months, catheter ablation was associated with significantly higher rates of freedom from AF compared with amiodarone (70% vs 34%; *P* < 0.001). Importantly, this superior rhythm control translated into clinically meaningful outcomes, including a significant reduction in all-cause mortality (8% vs 18%; *P* = 0.037) and unplanned hospitalizations (31% vs 57%; *P* < 0.001). The AATAC trial is particularly informative because it isolates the effect of ablation against the most commonly used AAD in this population. The findings underscore the limitations of pharmacological rhythm control, even with amiodarone, which is often considered the most effective AAD in HF but is associated with long-term toxicity and incomplete rhythm control. The improved outcomes observed with ablation suggest that durable rhythm control, rather than the strategy of rhythm control per se, is critical for prognostic benefit.

Additional mechanistic insights were provided by the CAMERA-MRI trial, which evaluated patients with AF and otherwise unexplained systolic dysfunction [24]. Catheter ablation resulted in substantial improvement in LVEF compared with medical rate control, with an absolute increase in ventricular function of approximately 18% versus 4.4% in the medical therapy group (*P* < 0.001). Importantly, the absence of ventricular fibrosis on cardiac magnetic resonance imaging was associated with particularly pronounced reverse remodeling after ablation, suggesting that early rhythm control may be especially beneficial in patients with potentially reversible myocardial dysfunction. These findings support the concept that AF itself may directly contribute to ventricular impairment and that restoration of sinus rhythm can reverse arrhythmia-mediated cardiomyopathy [25].

In contrast, the CABANA trial enrolled a broader AF population, including patients with and without HF, and compared catheter ablation with conventional medical therapy (rate or rhythm control). In the overall intention-to-treat analysis, CABANA did not demonstrate a statistically significant reduction in the primary composite endpoint of death, disabling stroke, serious bleeding, or cardiac arrest [26]. However, subgroup analyses of CABANA provide insights specific to patients with HF. In this population, catheter ablation was associated with improvements in survival, reductions in AF recurrence, and better quality of life compared with medical therapy [27]. Additionally, per-protocol and as-treated analyses demonstrated a more pronounced benefit of ablation, further supporting its efficacy when successfully implemented.

Taken together, AATAC, CAMERA-MRI, and CABANA complement the findings of CASTLE-AF by reinforcing several key concepts. First, catheter ablation achieves more durable and effective rhythm control than pharmacological therapy. Second, restoration of sinus rhythm may lead to significant reverse remodeling and recovery of ventricular function, particularly in arrhythmia-mediated cardiomyopathy. Third, improved rhythm control translates into reductions in hospitalizations and, in selected populations, mortality. Finally, the benefits of ablation appear consistent across different clinical settings, although the magnitude of effect may vary depending on patient characteristics, myocardial substrate, and HF stage.

## Catheter Ablation in Advanced HFrEF: CASTLE-HTx

The CASTLE-HTx trial represents a pivotal extension of prior evidence into the most advanced stage of HF, specifically patients with end-stage HF who were eligible for HTx or mechanical circulatory support. While earlier trials such as CASTLE-AF established the prognostic benefit of catheter ablation in HFrEF, patients with advanced HF had largely been excluded, leaving uncertainty regarding the applicability of rhythm control strategies in this high-risk population.

CASTLE-HTx evaluated the effect of catheter ablation in patients with end-stage HF eligible for HTx. Participants were 1:1 randomized to catheter ablation in addition to GDMT versus GDMT alone. The primary endpoint was a composite of all-cause mortality, LVAD implantation, or urgent HTx, events that reflect key milestones in the progression of advanced HF [8].

After a median follow-up of approximately 18 months, catheter ablation was associated with a substantial reduction in the primary endpoint (8% vs 30%, HR 0.24; *P* < 0.001), indicating a marked attenuation of disease progression. Importantly, this early benefit was not transient [8]. Extended follow-up data demonstrated a consistent and sustained treatment effect over time (20% vs 40%; *P* < 0.001) with separation of event curves maintained through approximately 3 years, supporting durability of benefit beyond the initial treatment phase [28, 29]. The magnitude and persistence of this effect are notable given the advanced disease stage of the enrolled population, in whom event rates are typically high and therapeutic options limited.

All-cause hospitalizations (78% vs 95%, HR 0.52; *P* < 0.001) as well as those related to worsening HF (45% vs 74%, HR 0.44; *P* < 0.001) have been reduced in patients randomized to catheter ablation [29]. Although hospitalization was not part of the primary endpoint, the observed reduction in clinical progression events suggests a stabilization of HF status that likely translates into fewer decompensations requiring hospital admission. Hospitalizations are a central determinant of clinical trajectory in advanced HF and reflect both disease severity and progression. In this population, recurrent HF decompensations are common and are independently associated with a stepwise increase in mortality risk. Thus, reduction in hospitalization burden represents a clinically meaningful therapeutic target. Traditional time-to-first-event analyses underestimate the impact of recurrent hospitalizations, particularly in advanced HF, where repeated admissions drive both morbidity and progression to advanced therapies. Consequently, interventions that reduce not only mortality, but also clinical deterioration events are of particular relevance.

Beside the survival benefit, functional recovery after 12 months has been observed in the ablation group with an LVEF improvement of 7.8% compared to 1.4% in the GDMT group. After 3 years in patients who did not reach the primary endpoint, improvements of LVEF were observed in both treatment groups, with LVEF increasing by 8.8% in the ablation group and 7.1% in the medical therapy group. Similarly, ablation procedures and optimization of HF medication occurred to a comparable degree among survivors in both arms. These findings suggest that, in patients who remain clinically stable, reverse remodeling and intensification of guideline-directed therapy are achievable irrespective of treatment strategy. However, catheter ablation was associated with a significantly greater reduction in AF burden (34% vs 11%; *P* < 0.001) and a lower prevalence of persistent AF (48% vs 72%; *P* = 0.002). This indicates that ablation provides superior rhythm control, which may facilitate clinical stabilization.

From a clinical perspective, these findings challenge the traditional paradigm that rhythm control is less relevant in advanced HF. Instead, they suggest that restoration of sinus rhythm, or meaningful reduction in AF burden, can favorably influence disease trajectory even in patients approaching end-stage HF. This is particularly relevant in transplant-eligible patients, where stabilization of clinical status may delay listing or reduce urgency. However, procedural safety is a critical consideration in this population. Despite the high-risk profile, catheter ablation in CASTLE-HTx demonstrated an acceptable safety profile, supporting feasibility in specialized centers.

### Risk Stratification – Which Patients Benefit Most?

Identification of patients most likely to benefit from catheter ablation remains a central aspect of rhythm control strategies in HFrEF. Both the CASTLE-HTx risk score and the Antwerp score provide important insights into patient selection and suggest that clinical benefit is closely linked to the severity of structural HF progression and AF burden.

A subanalysis of CASTLE-HTx identified three independent predictors of adverse outcomes: LVEF < 30%, New York Heart Association (NYHA) functional class ≥ III, and high AF burden [9]. Based on these variables, patients were stratified into lower- and higher-risk groups. Importantly, while event rates were highest in the high-risk group, these patients derived the greatest absolute benefit from catheter ablation compared with medical therapy alone. This finding suggests that advanced disease should not preclude ablation; rather, patients at highest risk of progression may benefit most.

Complementary insights are provided by the Antwerp score, a clinical risk stratification tool developed to predict response to catheter ablation in patients with AF and HFrEF [30]. The score integrates four markers (wide QRS duration > 120 ms, known HF etiology, severe dilated left atrium, and paroxysmal AF), which have been associated with a lower likelihood of reverse remodeling and long-term rhythm success after ablation(31). Lower Antwerp scores were associated with greater improvement in ventricular function and clinical outcomes, whereas higher scores reflected more advanced myocardial disease and reduced response to catheter ablation.

Taken together, these risk models support a more individualized approach to patient selection. While the CASTLE-HTx model identifies patients at highest risk who may derive the greatest absolute prognostic benefit, the Antwerp score provides additional insight into the extent of structural remodeling and potential reversibility. Both approaches emphasize that AF burden alone is insufficient for treatment decisions and that comprehensive assessment of HF severity and myocardial substrate is essential when considering catheter ablation in advanced HF stages. However, the identified parameters are readily available in clinical practice, supporting pragmatic risk stratification and facilitating early referral for catheter ablation within advanced HF and transplant evaluation pathways.

### CASTLE-HTx Subanalysis: Cost-Effectiveness of Catheter Ablation

Beyond its clinical efficacy, a recent subanalysis of the CASTLE-HTx trial provides important insights into the economic implications of catheter ablation in patients with end-stage HFrEF and AF. Using a cohort-based Markov model informed by the three-year outcomes of CASTLE-HTx, lifetime costs, life-years (LYs), and quality-adjusted life-years (QALYs) were projected for catheter ablation in combination with GDMT compared with GDMT alone.

In lifetime projections, catheter ablation was associated with a mean survival gain of approximately 1.7 life-years per patient at an incremental cost of roughly €12,000, resulting in an incremental cost-effectiveness ratio (ICER) of approximately €7,300 per life-year gained. When adjusted for quality of life, the ICER was estimated at €8,600 per QALY in lifetime projections and €30,400 per QALY at 36 months—both well below commonly accepted willingness-to-pay thresholds of €50,000 per QALY. Within the first 3 years, the observed survival benefit translated into a gain of 0.34 life-years, reflecting a reduction in mortality and HF progression events. Notably, the economic benefit emerged early and remained consistent across multiple sensitivity analyses.

These findings indicate that catheter ablation is not only clinically effective but also economically favorable in patients with advanced HF referred for transplant evaluation. By reducing mortality and delaying progression to resource-intensive therapies such as LVAD implantation and HTx, catheter ablation represents a cost-effective strategy within advanced HF management pathways.

## Mechanistic Insights Beyond AF Burden Reduction in Advanced HF

The clinical benefits of catheter ablation in patients with advanced HF stages extend beyond simple reduction of AF burden. Additional factors, including regression from persistent to paroxysmal AF phenotype, reduction in mean heart rate, and decreased AAD usage, contribute to the observed improvements in outcomes. In particular, improved heart rate variability and more physiological rate control during daily activity may mitigate cellular calcium overload, reduce neurohumoral activation, and thereby promote reverse remodeling [32].

**Fig. 3:**
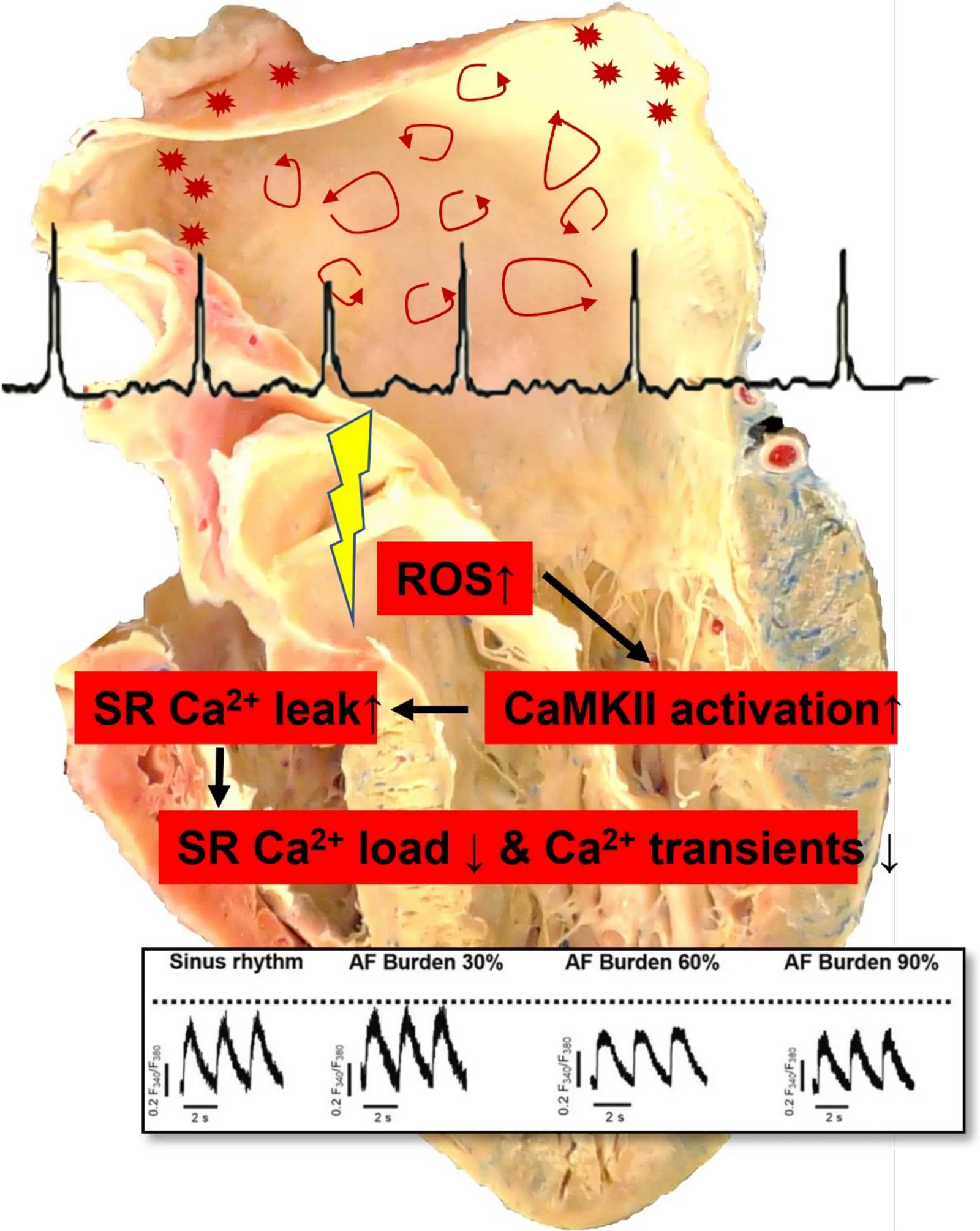
**Mechanistic insights of atrial fibrillation in advanced heart failure.** The detrimental remodeling of atrial fibrillation (AF) on the left ventricle. AF causes excessive reactive oxygen species (ROS) formation that actives Ca2 + –calmodulin-dependent protein kinase II (CaMKII) thereby causing diastolic sarcoplasmic Ca2 + loss and depressed Ca2 + transients. Experimental data indicate, that remodeling of ventricular remodeling cardiomyocytes occurs between an AF burden of 30–60% [34], [38]

Translational data provide further mechanistic support for these observations. Human ventricular cardiomyocytes exposed to AF-like electrical stimulation develop hallmark features of HF, including impaired calcium (Ca^2^⁺) handling and contractile dysfunction (Fig. 3). Specifically, AF-like stimulation leads to disturbed Ca^2^⁺ transients, which are essential for excitation–contraction coupling. These alterations are mediated, at least in part, by increased reactive oxygen species (ROS) formation, resulting in activation of Ca^2^⁺/calmodulin-dependent protein kinase II (CaMKII). This signaling cascade promotes sarcoplasmic reticulum Ca^2^⁺ leak and reduces intracellular Ca^2^⁺ availability, ultimately impairing myocardial contractility [33]. Importantly, these detrimental effects appear to be arrhythmia burden-dependent. An AF burden of approximately ≥ 60% has been associated with significant abnormalities in calcium handling and reduced sarcomere organization in human cardiomyocytes [34][38]. These findings suggest a threshold effect, beyond which AF significantly impacts ventricular structure and function negatively. These mechanistic insights provide a translational rationale for the clinical benefits observed in trials such as CASTLE-AF and CASTLE-HTx. Catheter ablation may improve outcomes in advanced HF stages not only by restoring sinus rhythm but also by reducing AF burden sufficiently to reverse key cellular and molecular processes driving HF progression.

## Emerging Technologies: Pulsed Field Ablation

PFA represents a novel, non-thermal ablation modality based on irreversible electroporation, enabling myocardial tissue selectivity while largely sparing surrounding structures such as the esophagus, phrenic nerve, and pulmonary veins [35]. Early clinical data suggest that PFA is associated with high acute procedural success and a favorable safety profile compared with conventional thermal energy sources [36]. An additional advantage of PFA is the potential for substantially shorter procedure times due to rapid lesion formation and simplified workflows [37]. In patients with advanced HF, in whom prolonged procedures may be associated with hemodynamic instability and increased periprocedural risk, reduced procedure duration may be clinically relevant. This may facilitate broader applicability of catheter ablation in high-risk populations who were previously considered suboptimal candidates. While long-term outcome data remain limited, the combination of improved safety, procedural efficiency, and reproducibility suggests that PFA may further expand the role of catheter ablation in patients with AF and advanced HF [21].

## Clinical Implications and Future Directions

Catheter ablation improves survival and reduces progression to advanced therapies in HFrEF. High-risk patients derive substantial benefit, supporting integration into advanced HF management pathways. The cumulative evidence from randomized trials, particularly CASTLE-AF and CASTLE-HTx, supports a fundamental shift in the management of patients with concomitant AF and HFrEF. AF in the setting of HF should no longer be regarded as a comorbidity but rather as a key driver of disease progression and adverse outcomes. This conceptual transition has direct therapeutic consequences. First, catheter ablation has emerged as a disease-modifying intervention in HFrEF. The survival benefit demonstrated in CASTLE-AF establishes ablation as more than a symptomatic therapy, indicating that effective rhythm control can alter long-term prognosis. This is particularly relevant given the historical limitations of AAD-based strategies. Second, the findings of CASTLE-HTx extend this paradigm to patients with advanced HF stages. In this high-risk population, catheter ablation significantly reduces the combined risk of death from any cause, LVAD implantation, and urgent HTx. These outcomes represent critical milestones in HF progression, underscoring that ablation can influence not only symptoms but also the trajectory toward advanced therapies. Third, risk stratification analyses suggest that patients with more advanced disease—characterized by severely reduced LVEF, higher New York Heart Association (NYHA) functional class, and greater AF burden—may derive the greatest absolute benefit from catheter ablation. This challenges the traditional reluctance to pursue rhythm control in advanced HF and instead supports a more proactive approach in carefully selected high-risk patients.

Taken together, these data argue for earlier consideration of catheter ablation within the HF treatment algorithm. Rather than reserving ablation as a last-line therapy, it should be integrated into a comprehensive management strategy aimed at modifying disease progression. These findings have implications for advanced HF care pathways, where transplant programs should consider incorporating AF ablation into their evaluation and treatment algorithms, particularly in patients with significant AF burden. Such integration may stabilize clinical status, delay progression to mechanical circulatory support or HTx, and ultimately improve survival.

In parallel, the ongoing CASTLE-HFpEF study (ClinicalTrials.gov number: NCT 07254455) will further expand this concept by evaluating the impact of early catheter ablation in patients with AF and HF with preserved ejection fraction, potentially extending the disease-modifying role of rhythm control across the broader HF spectrum.

## Conclusion

Catheter ablation has transformed the management of AF in HFrEF with evidence of reduced mortality, hospitalizations, and progression to advanced therapies, including those with advanced HF stages. Earlier intervention should be considered following individualized assessment, with management within experienced electrophysiology centers. In appropriately selected patients with AF and advanced HF: don’t wait—ablate.

## Key References

- Marrouche NF, Brachmann J, Andresen D, Siebels J, Boersma L, Jordaens L, et al. Catheter Ablation for Atrial Fibrillation with Heart Failure. New England Journal of Medicine. 2018;378(5):417–27. 
  - ○ A randomized trial demonstrating the benefit of catheter ablation in HFrEF patients with atrial fibrillation.
- Sohns C, Fox H, Marrouche NF, Crijns H, Costard-Jaeckle A, Bergau L, et al. Catheter Ablation in End-Stage Heart Failure with Atrial Fibrillation. N Engl J Med. 2023;389(15):1380–9. 
  - ○ A randomized trial demonstrating the benefit of catheter ablation in advanced HF patients with atrial fibrillation.
- Moersdorf M, Tijssen JGP, Marrouche NF, Crijns H, Costard-Jaeckle A, Bergau L, et al. Prognosis of patients in end-stage heart failure with atrial fibrillation treated with ablation: Insights from CASTLE-HTx. Heart Rhythm. 2024. 
  - ○ A risk stratification demonstrating advanced HF patients who benefit more likely from catheter ablation.
- Pabel S, Knierim M, Stehle T, Alebrand F, Paulus M, Sieme M, et al. Effects of Atrial Fibrillation on the Human Ventricle. Circulation research. 2022;130(7):994–1010.
  - ○ The manuscript highlights molecular mechanisms and effects of atrial fibrillation on ventricular function.

## Data Availability

No datasets were generated or analysed during the current study.

## Abbreviations

AAD: Antiarrhythmic drug
AF: Atrial fibrillation
Ca^2^⁺: Calcium
GDMT: Guideline-directed medical therapy
HTx: Heart transplantation
HF: Heart failure
HFpEF: Heart failure with preserved ejection fraction
HFrEF: Heart failure with reduced ejection fraction
HR: Hazard ratio
ICER: Incremental cost-effectiveness ratio
LVAD: Left ventricular assist device
LVEF: Left ventricular ejection fraction
LY: Life-years
PFA: Pulsed field ablation
QALY: Quality-adjusted life-years
RAAS: Renin–angiotensin–aldosterone system
